# Early and dynamic alterations of Th2/Th1 in previously immunocompetent patients with community-acquired severe sepsis: a prospective observational study

**DOI:** 10.1186/s12967-019-1811-9

**Published:** 2019-02-27

**Authors:** Ming Xue, Jianfeng Xie, Ling Liu, Yingzi Huang, Fengmei Guo, Jingyuan Xu, Yi Yang, Haibo Qiu

**Affiliations:** Department of Critical Care Medicine, Zhongda Hospital, School of Medicine, Southeast University, Nanjing, 210009 China

**Keywords:** Community-acquired severe sepsis, T helper cells, Mortality, ICU-acquired infection

## Abstract

**Background:**

T helper (Th) cells regulate sepsis processes, including primary pathogen clear and secondary pathogen defence. The objectives of this study were to determine the early and dynamic alterations of Th1 and Th2 populations to community-acquired severe sepsis upon onset among previously immunocompetent patients and whether it was related to clinical outcomes.

**Methods:**

This prospective observational cohort study was conducted at a general intensive care unit (ICU) of a tertiary teaching hospital in China. Immunocompetent patients with community-acquired severe sepsis within 24 h upon onset were included as septic group. Healthy volunteers and critically ill patients without severe sepsis were recruited as controls. Whole blood was collected on D0, 3rd day (D3) and 7th day (D7) for septic group and once upon enrollment for controls. Th1 and Th2 populations were measured by flow cytometry and assessed for associations with 28-day mortality using cox proportional hazard models. Associations of dynamic alterations of Th cell subpopulations with clinical outcomes were investigated.

**Results:**

This study demonstrated that community-acquired severe sepsis patients (n = 71) had increased Th2/Th1 and Th2 populations, compared to healthy controls (n = 7) and critically ill patients without severe sepsis (n = 7) at admission. Among the septic cohort, values of Th2/Th1 were significantly higher in non-survivors than survivors on D0 (p = 0.04), D3 (p < 0.001) and D7 (p < 0.001). Patients with persistently increasing Th2/Th1 demonstrated the highest mortality (47.1%) and incidence of ICU-acquired infections (64.7%).

**Conclusions:**

Th2/Th1 was markedly up-regulated with Th2 dominance upon community-acquired severe sepsis onset among previously immunocompetent patients and its persistently dynamic increase was associated with ICU-acquired infections and 28-day death.

*Trial registration* Institutional Ethics Committee of Zhongda Hospital, 2014ZDSYLL086, registered in June 2014-prospectively registered; ClinicalTrials.gov, NCT02883218, registered on 25 Aug 2016-retrospectively registered, https://www.clinicaltrials.gov/ct2/show/NCT02883218?cond=NCT02883218&rank=1

**Electronic supplementary material:**

The online version of this article (10.1186/s12967-019-1811-9) contains supplementary material, which is available to authorized users.

## Background

Sepsis is characterized by a dysregulated host response to infection, which brings organ failure and death [1, 2]. Many trials of immunomodulatory drugs failed to improve patient outcomes [3, 4]. Although many factors may have contributed to negative findings, clinical criteria do not adequately delineate patients who benefit from specific therapies [5, 6].

Currently, sepsis-induced immunosuppression is prevailing in sepsis pathophysiology, during which the adaptive immune system is characterized with cell apoptosis, cellular exhaustion and hypo-responsiveness [7]. Despite active research in sepsis-induced immune dysfunction and immuno-therapy, there are several important gaps between our understanding and practice. First, the time of sepsis-induced immunosuppression remains controversial. Pro- as well as anti-inflammatory may cycle through each phase multiple times over the sepsis course [8, 9]. When the immune system alters during sepsis process needs a rational explanation. Secondly, immune status at the cellular level rather than inflammatory cytokine levels is lack of study, which might differ clinical outcomes. Lastly, the immune status’ alteration after sepsis rather than a single value at a given time point matters [10]. Dynamic adaptive immune status monitor remains lack.

T helper cells as parts of adaptive immunity, drive and control immune responses. Th1 cells release mainly proinflammatory cytokine interferon (IFN)-γ inducing phagocytosis and intracellular killing of microbes while Th2 cells drive specific responses for extracellular pathogens and resolute cell-mediated inflammation by secreting the mainly anti-inflammatory cytokine interleukin (IL)-10 and IL-4. In such cases, Th1 and Th2 cells cross-regulate one another and the balance is important for clearing the infection [11, 12]. The imbalance with Th2 dominant would lead to secondary infections, viral reactivations, and an inability to clear the initial infection, which has been proved in autoimmune diseases [13]. Predominating Th2 in patients with sepsis rather than non-sepsis was proved in Ferguso study [14]. However, the timing of Th2 cell subset predominance and the effect of its dynamic alterations on risk of infection and mortality remains unknown upon sepsis onset. Clarifying its alterations and the relationship with clinical outcomes in severe sepsis will help stratify patients when designing an adaptive immune-targeted therapy in a time-dependent manner.

In this study, we included previously immunocompetent patients with new-onset community-acquired severe sepsis. We characterized the balance of the host adaptive immune response at various time points following sepsis onset by using Th2/Th1 and aimed to investigate the dynamic alterations of Th1 and Th2 populations to community-acquired severe sepsis upon onset and whether it was related to clinical outcomes.

## Methods

### Study design and population

We conducted a prospective cohort study in a 60-bed general intensive care unit (ICU) of a tertiary teaching hospital in Nanjing, China. This study was approved by the Institutional Ethics Committee of Zhongda Hospital (Approval Number: 2014ZDSYLL086) before enrolment of the first participant and was in full compliance with Declaration of Helsinki. The study was retrospectively registered with ClinicalTrials.gov, NCT02883218. All the participants provided written informed consent.

Eligible patients between 18 and 90 years old that admitted to the ICU with a diagnosis of community-acquired severe sepsis according to criteria of the American College of Chest Physicians/Society of Critical Care Medicine [15] within 24 h after sepsis-induced organ dysfunction recognition, were recruited as septic group between 18 September, 2014 and 28 September, 2016. Patients were excluded if they had tumors, hematological or immunological disease, or treatment with chemotherapy agents or corticosteroids within 6 months prior to hospitalization.

Heathy volunteers coming for physical examination in and patients admitted to the ICU without a diagnosis of severe sepsis, between 18 and 90 years old, without tumors, hematological or immunological disease, or treatment with chemotherapy agents or corticosteroids within 6 months prior were recruited as healthy control and ICU control, respectively. The sample size of health controls and ICU controls would be calculated with a power of 0.80 and alpha of 0.05 by Power Analysis and Sample Size (PASS) software (PASS 2008. citation: Hintze J (2008). NCSS, LLC. Kaysville, Utah, USA) [16], based on recruitment of severe sepsis and the difference of Th2/Th1 between severe sepsis vs. health controls (2.17 vs. 0.26) and severe sepsis vs. ICU controls (2.17 vs. 0.4), as the Ferguso study showed [14].

### Outcomes and definitions

We aimed to determine alterations of Th1 and Th2 populations and their associations with 28-day prognosis. Secondly, we stratified the septic patients according to alterations of Th2/Th1 within 1 week: (1) the early recovery group consisted of septic patients with overall decreasing Th2/Th1; (2) the late recovery group with Th2/Th1 starting to decrease after D3; (3) the non-recovery group with an overall increasing Th2/Th1, and then assessed their associations with clinical outcomes.

The onset of severe sepsis was defined as the time of diagnosis of sepsis-induced organ dysfunction. The first 24-h period after enrolment was to be D0; the next 24-h period was D1, etc. We followed the Berlin criteria for acute respiratory distress syndrome (ARDS) [17]. Acute kidney injury (AKI) was defined according to the kidney disease improving global outcomes (KDIGO) criteria [18]. Acute gastrointestinal injury (AGI) was defined according to the European Society of Intensive Care Medicine (ESICM) Working Group [19]. The occurrence of ICU-acquired infections, defined as a first occurrence of bloodstream infection, or pneumonia with a post hoc plausibility of infection rated at least possible according to strict definitions [20] within 28 days.

### Procedures

For septic patients, baseline demographics including age, gender, suspicious infection sites, presence of comorbidities, smoking and alcohol use histories, acute physiology and chronic health evaluation (APACHE) II score and sequential organ failure assessment (SOFA) score [21], and clinical data including systemic inflammatory indicators, such as temperatures, heart rates and count of white blood cell (WBC), as well as absolute lymphocyte cell (ALC), procalcitonin (PCT) and hypersensitive C-reactive protein (hs-CRP) on D0, D3 and D7, occurrence of complicated organ dysfunction and ICU-acquired infection, and prognosis within 28 days were collected from documentations recorded by the treating physician. For ICU controls, baseline demographics were recorded that included age, sex, primary diagnosis, presence of comorbidities, smoking and alcohol use histories, APACHE II score, SOFA score and levels of WBC, ALC, PCT as well as hs-CRP upon enrolment.

**Fig. 1:**
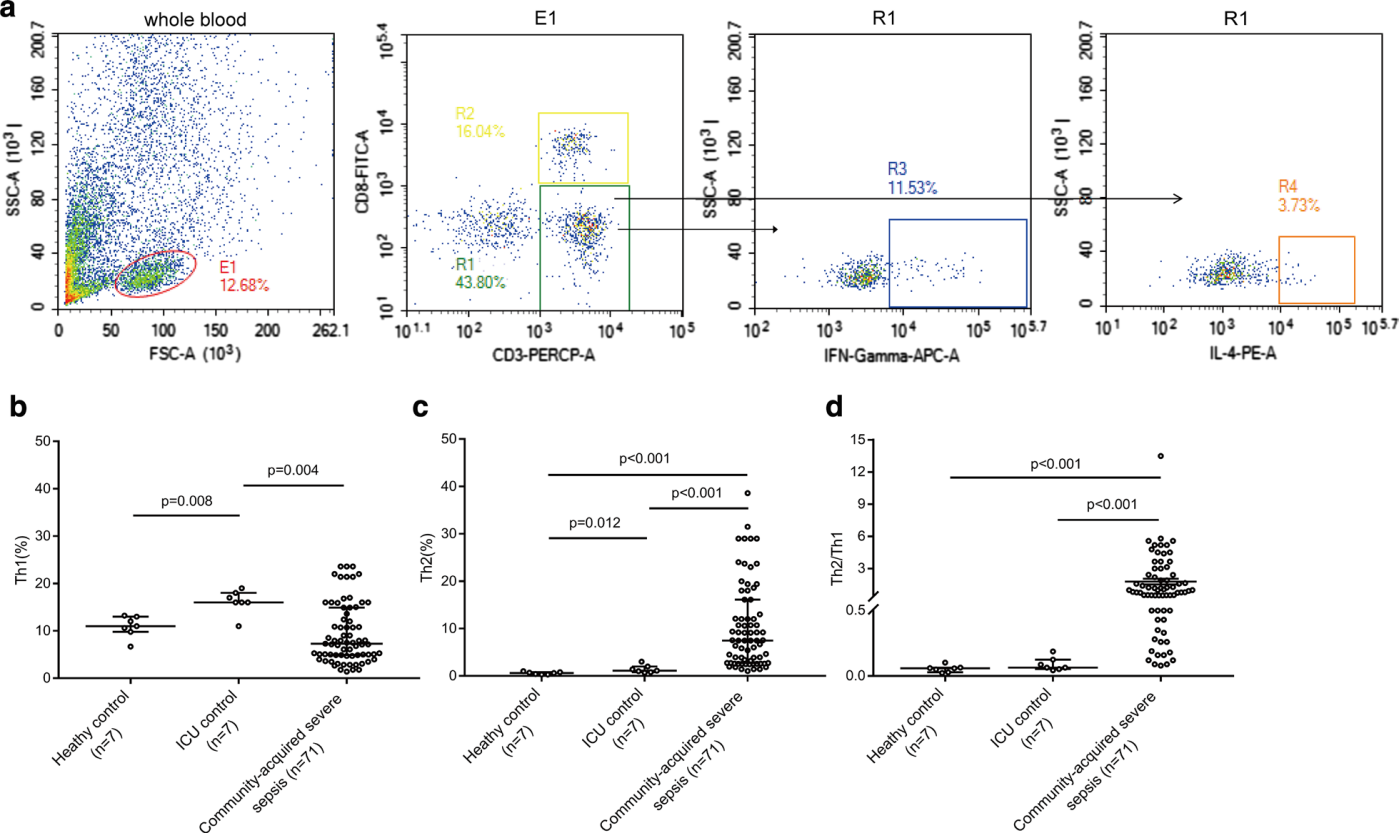
Values of Th1, Th2 and Th2/Th1 in heathy control, ICU control and septic groups upon enrollment. **a** Gating strategy used for flow cytometric analysis. CD3 positive (CD3+) cells were analysed and further specified according to size/granularity in E1. R1 in green represents CD3+ CD8− T cells, which approximately equal to CD4+ T cells while R2 in yellow stands for CD8+ T cells. Th1 cells are defined as INF-gamma+ cells in CD3–CD8+ cells as R3 shows in blue. Th2 cells are defined IL4+ cells in CD3–CD8+ cells as R4 shows in orange. Values of Th1 (**b**), Th2 (**c**) and Th2/Th1 (**d**) upon enrollment in heathy control, ICU control and septic groups are presented as scatter dot plots with lines for median value and quartiles

Whole blood was collected in heparin sodium anticoagulant tubes on D0, D3 and D7 for septic group and once upon enrolment for controls. T lymphocyte subpopulations were measured by flow cytometry as descripted in Additional file 1. T helper cells gating strategy used for flow cytometric analysis were shown in Fig. 1a. Data were analysed in FlowJo version 10 (Ashland, OR, USA). Levels of IFN-γ, IL-4 and IL-10 in plasma collected upon enrolment in healthy control, ICU control and severe sepsis patients were determined using commercial ELISA kits: EH008-96, EH003-96, EH006-96 (ExCell Bio, Taicang, China) according to manufacturer’s instructions. For severe sepsis group, concentrations of plasma IL-4, IL-10 and INF-γ were consecutively measured on D3 and D7.

### Statistical analysis

Data of patients that could not get consecutive T helper cell measurements on D0, D3 and D7 were excluded. T helper cells populations were expressed as numbers in CD3+ CD8− T lymphocytes (%). Data were analysed using SPSS Version 23 (IBM, Chicago, Ill, USA) and GraphPad PRISM Version 5.3 (San Diego, CA, USA). Descriptive statistics, including the mean ± standard deviation (SD), median (interquartile range [IQR] defined as the 25th and 75th percentile), median ± mean squared error (SEM) and mean ± SEM were used as appropriate. Variables that failed tests of normality were presented and analysed in a nonparametric fashion. Comparisons of characteristics among cohorts, between 28-day survivors and non-survivors, among subgroups according to Th2/Th1 alterations were performed using unpaired t tests, Mann–Whitney U tests, or Chi squared tests, as appropriate. All tests were two-tailed, and a value of p < 0.05 was considered statistically significant. Correlations between Th1 populations and plasma INF-γ levels, and Th2 populations and plasma IL-4 levels were analysed with Pearson correlation test and presented as R square, when R square is more than 0.64 with a p value < 0.05, it would be regarded as a significant correlation.

Univariate analysis of cox regression was performed initially, using SPSS, on the septic cohort data (n = 71) to identify variables that were independently associated with 28-day mortality. The variables entered included demographics, severity score, inflammatory and immune indicators including WBC, ALC, Th1 and Th2 populations, PCT as well as hs-CRP and alterations of these indicators. Variables identified with a threshold of p < 0.05 were investigated for associations with 28-day mortality in a cox-proportional hazards model. Specific cox models were conducted for each variable that were mathematically coupled or collinear with each other, such as WBC and ALC, and T helper population and its alterations on D0, D3 and D7 respectively. Hazard ratios were provided for each variable included in the final model with 95% confidence intervals (CIs).

Receiver-operating characteristic (ROC) curve analysis was performed to assess the ability of the severity scores, inflammatory and immune indicators as well as its alterations to predict mortality. CIs on areas under the curves (AUCs) were calculated using nonparametric assumptions. The best threshold was determined using the Youden index with sensitivity and specificity for variables with p < 0.05 and AUC above 0.5. Kaplan–Meier analysis was performed to determine the survival lifetimes of subgroups for 28-day survival, and a log-rank test was used to compare curves.

## Results

A total of 71 patients with community-acquired severe sepsis patients completed the study and were analyzed for septic group (Additional file 2: Figure S1). The baseline characteristics were shown in Table 1. The sample size of ICU controls and healthy controls was both 3 based on number of 71 in community-acquired severe sepsis by PASS software with a power of 0.80. Considering the age and gender distribution in community-acquired severe sepsis patients, we finally included seven ICU control patients and seven healthy control patients (Table 1).

**Table 1:** Characteristics of enrolled patients

Characteristic	Healthy control n = 7	ICU control n = 7	Community-acquired severe sepsis n = 71			
			Overall	28-Day survivors	28-Day non-survivors	p
			n = 71	n = 53 (74.6%)	n = 18 (25.4%)	
Age (years)	57.32 ± 23.27	69.00 ± 19.87	71.72 ± 15.54	71.85 ± 17.21	71.72 ± 10.23	ns
< 60, n (%)	2	2	15	12	3	
61–70, n (%)	1	1	9	5	4	
71–80, n (%)	2	2	23	15	8	
81–90, n (%)	2	2	23	22	3	
Gender (male/female)	4/3	4/3	45/26	36/17	9/9	ns
Admission sources (n, %)						ns
Emergency	–	1 (14.3)	56 (78.9)	40 (75.5)	16 (88.9)	
Internal dept.	–	2 (28.6)	6 (8.5)	6 (11.3)	0	
Surgical dept.	–	4 (57.1)	9 (12.7)	7 (13.2)	2 (11.1)	
Presumed infection site (n, %)						ns
Lung	–	–	50 (70.4)	36 (67.92)	14 (77.7)	
Abdomen	–	–	11 (15.5)	8 (15.09)	3 (16.7)	
Cardiovascular system	–	–	3 (4.2)	3 (5.66)	0	
Bone or soft tissue	–	–	1 (1.4)	1 (1.89)	0	
Urinary tract	–	–	6 (8.5)	5 (9.43)	1 (5.6)	
Aetiology^a^						
Bacteria	–	–	32 (45.1)	21 (39.6)	11 (61.1)	ns
Virus	–	–	5 (7.0)	3 (5.7)	2 (11.1)	ns
Others or undetected	–	–	34 (47.9)	29 (54.7)	5 (27.8)	ns
Comorbidities (n, %)						
Hypertension	0	3 (42.9)	38 (53.5)	28 (52.8)	10 (55.6)	ns
Diabetes	0	3 (42.9)	20 (28.2)	13 (24.5)	7 (38.9)	ns
Chronic cardiac dysfunction	0	2 (28.6)	27 (38.0)	20 (37.7)	7 (38.9)	ns
Cerebrovascular disease	0	0	28 (39.4)	22 (41.5)	6 (33.3)	ns
Chronic renal dysfunction 0	0	0	5(7.0)	4(7.5)	1(5.6)	ns
Smoker						ns
Never	6 (85.7)	6 (85.7)	47 (66.2)	33 (62.3)	14 (77.8)	
Previous (stopped > 3 months)	0 (0)	1 (14.3)	14 (19.7)	11 (20.8)	3 (16.7)	
Former (stopped ≤ 3 months)	0 (0)	0 (0)	0 (0)	0 (0)	0 (0)	
Current	1 (14.3)	0 (0)	10 (14.1)	9 (16.9)	1 (5.6)	
Alcohol use						ns
Never or daily intake ≤ 50 g of pure alcohol	7 (100)	6 (85.7)	64 (90.1)	46 (86.8)	18 (100)	
Previous (daily intake > 50 g of pure alcohol, stopped > 3 months)	0 (0)	1 (14.3)	3 (4.2)	3 (5.7)	0 (0)	
Former (daily intake > 50 g of pure alcohol, stopped ≤ 3 months)	0 (0)	0 (0)	0 (0)	0 (0)	0 (0)	
Current intake (> 50 g of pure alcohol)	0 (0)	0 (0)	4 (5.6)	4 (7.5)	0 (0)	
Severity scores						
APACHE II	–	19.51 ± 5.31	20.55 ± 5.95^#^	19.51 ± 5.31	23.38 ± 6.19	0.04
SOFA D0	–	6.00 ± 2.39	9.91 ± 3.11^#^	9.70 ± 3.21	10.44 ± 2.93	ns
D3	–	–	8.11 ± 3.66	7.43 ± 3.35	9.5 ± 4.01	0.04
D7	–	–	7.02 ± 3.29	6.24 ± 3.01	9.13 ± 3.31	0.01
Inflammatory and immune indicators						
D0 (upon enrollment)						
WBC (/μl)	6.31 [5.23, 7.54]	13.89 [8.57,16.22]	13.66 [8.89, 20.14]	13.66 [8.59, 18.92]	14.61 [8.89, 22.23]	ns
ALC (/μl)	2.74 [1.99, 2.86]	1.07 [0.96, 2.22]	0.59 [0.52, 0.90]^#^	0.59 [0.51, 0.99]	0.52 [0.51, 0.66]	ns
hs-CRP (mg/l)	–	6 [0.81, 119]	94.60 [44, 136]^#^	96.50 [65.85, 146.50]	85.20 [33.88, 126.30]	ns
PCT (μg/l)	–	0.09 [0.05, 2.05]	1.18 [0.34, 12.82]^#^	0.98 [0.30,17.79]	1.27 [0.47, 1.93]	ns
Treg (%)	1.77 [1.11, 2.56]	1.52 [1.07, 1.77]	2.14 [1.44, 3.91]	2.14 [1.49, 3.54]	1.69 [0.39, 5.40]	ns
D3						
WBC (/μl)	–	–	9.78 [7.35, 15.29]	9.47 [7.17, 9.47]	14.07 [7.68, 19.59]	0.03
ALC (/μl)	–	–	0.93 [0.55, 1.30]	0.99 [0.59, 1.42]	0.76 [0.55, 0.83]	0.02
hs-CRP (mg/l)	–	–	63.5 [36.6, 107]	65.8 [41.5, 110]	45 [24, 105.9]	ns
PCT (μg/l)	–	–	0.87 [0.27, 2.53]	0.90 [0.26, 3.53]	0.53 [0.26, 2.15]	ns
Treg (%)	–	–	1.94 [1.39, 2.86]	1.97 [1.41, 2.87]	1.47 [0.35, 2.55]	0.05
D7						
WBC (/μl)	–	–	8.21 [6.08, 12.02]	7.98 [5.90, 9.06]	13.36 [6.30, 15.90]	0.02
ALC (/μl)	–	–	0.97 [0.66, 1.28]	0.99 [0.78, 1.29]	0.62 [0.47, 1.11]	0.02
hs-CRP (mg/l)	–	–	43.0 [14.75, 78.8]	46.85 [15.98, 81.4]	32.6 [13.45, 58.28]	ns
PCT (μg/l)	–	–	0.28 [0.20, 0.58]	0.21 [0.20, 0.46]	0.69 [0.22, 1.37]	ns
Treg (%)	–	–	1.38 [1.11, 2.17]	1.37 [1.11, 1.89]	2.17 [0.60, 3.91]	ns

APACHE, acute physiology and chronic health evaluation; SOFA, sequential organ failure assessment; WBC, white blood count; ALC, absolute lymphocyte count; hs-CRP, high-sensitivity C-reactive protein; PCT, procalcitonin; Treg (%), percentage of regulatory T cells in CD4 positive T cells

^a^Referred to any pathogen detected within 7 days upon enrolled; ^#^ Significant difference with p < 0.05 compared to ICU control. Chi square tests were used for categorical data, Student’s t-test for normally distributed continuous data and Mann–Whitney-U tests for nonnormally distributed data. Mean and standard deviations are shown for age and severity scores and medians and interquartile ranges are for all inflammatory and immune indicators

### Clinical presentation and outcomes

APACHE II and SOFA scores at admission in septic patients were significantly higher than that in ICU controls, indicating a higher level of severity. By 28 days, 18 septic patients (25.4%) had died (Table 1).

Compared to septic patients who survived, patients who died presented a higher APACHE II at admission and SOFA score on D3 and D7. APACHE II score at admission and SOFA scores on D3 and D7 was associated with mortality in univariate analysis of septic cohort (Additional file 3: Table S2).

**Table 2:** Characteristics of patients in subgroups stratified by alterations of Th2/Th1

Characteristic	Early recovery (n = 35)	Late recovery (n = 19)	Non-recovery (n = 17)	p
Age, mean ± SD, years	72.0 ± 16.8	75.7 ± 11.4	67.5 ± 16.2	ns
Gender (male/female)	25/10	10/9	10/7	ns
Admission sources, n (%)				0.03
Emergency	23 (65.7)	18 (94.7)	16 (94.1)	
Internal dept.	5 (14.3)	1 (5.3)	0	
Surgical dept.	7 (20)	0	2 (11.8)	
APACHE II, mean ± SD	20.9 ± 5.3	19.5 ± 4.2	20.8 ± 8.0	
SOFA, mean ± SD				
D0	9.3 ± 2.1	11.2 ± 3.1	9.6 ± 4.5	ns
D3	6.3 ± 2.6	10.8 ± 3.3	8.2 ± 3.8	< 0.001
D7	6.0 ± 3.2	8.0 ± 2.5	7.9 ± 4.0	ns
Presumed infection site, n (%)				ns
Lung	22 (62.9)	17 (89.5)	11 (64.7)	
Abdomen	6 (17.1)	2 (10.5)	3 (17.6)	
Cardiovascular system	2 (5.7)	0	1 (5.9)	
Bone or soft tissue	0	0	1 (5.9)	
Urinary tract	5 (14.3)	0	1 (5.9)	
Comorbidities, n (%)				
Hypertension	20 (57.1)	11 (57.8)	7 (41.2)	ns
Diabetes	8 (22.9)	6 (31.6)	6 (35.3)	ns
Chronic cardiac dysfunction	11 (31.4)	12 (63.2)	4 (23.5)	0.03
Cerebrovascular disease	11 (31.4)	9 (47.4)	8 (47.1)	ns
Chronic renal dysfunction	2 (5.7)	2 (10.5)	1 (5.9)	ns
Smoker				ns
Never	23 (65.7)	13 (68.4)	11 (64.7)	
Previous (stopped > 3 months)	8 (22.9)	4 (21.1)	2 (11.8)	
Former (stopped ≤ 3 months)	0 (0)	0 (0)	0 (0)	
Current	4 (11.4)	2 (10.5)	4 (23.5)	
Alcohol use				ns
Never or daily intake ≤ 50 g of pure alcohol	31 (88.6)	18 (94.7)	15 (88.2)	
Previous (daily intake > 50 g of pure alcohol, stopped > 3 months)	2 (5.7)	1 (5.6)	0 (0)	
Former (daily intake > 50 g of pure alcohol, stopped ≤ 3 months)	0 (0)	0 (0)	0 (0)	
Current intake (> 50 g of pure alcohol)	2 (5.7)	0 (0)	2 (11.8)	

Chi square tests were used for categorical data, Student’s t-test for normally distributed continuous data and Mann–Whitney-U tests for nonnormally distributed data

APACHE, acute physiology and chronic health evaluation; SOFA, sequential organ failure assessment

### Inflammatory and immune indicators in community-acquired severe sepsis vs. controls

Compared to healthy controls, community-acquired severe sepsis patients had increased Th2 populations (p < 0.001) along with higher Th2/Th1 (p < 0.001) at admission (Fig. 1b, c). Compared to ICU controls, community-acquired severe sepsis patients had higher levels of PCT (p = 0.008), hs-CRP (p = 0.020), lower Th1 population (p = 0.004) and higher T helper 2 cells (p < 0.001) along with higher Th2/Th1 (p < 0.001) at admission (Table 1 and Fig. 1). Absolute counts of lymphocyte were lower (p = 0.004) whereas white blood cell counts were similar (Table 1). Levels of plasma IFN-γ, IL-4 and IL-10 in blood serum collected upon enrolment in healthy control, ICU control and severe sepsis patients were measured. Compared with the healthy control, the IFN-γ levels of septic group were significantly higher (p < 0.05). There was no significant difference of IFN-γ between ICU controls and septic groups. In addition, IL-4 and IL-10 levels were higher in septic group than that of ICU controls and healthy controls (p < 0.05, see Additional file 3: Table S1).

### Inflammatory and immune indicators in community-acquired severe sepsis who died vs. survivors

As Table 1 showed, inflammatory indicators such as PCT and hs-CRP were markedly elevated and gradually decreased thereafter. Patients who died within 28 days had lower levels of ALC on D3 (p = 0.02) and D7 (p = 0.02) than those of 28-day survivors with a gradual recovery within a week. By univariate analysis, ALC on D7 was a protective factor of 28-day prognosis (p = 0.013) while levels of WBC on D3 and D7 were risk factors (p = 0.005 and 0.001, respectively).

**Fig. 2:**
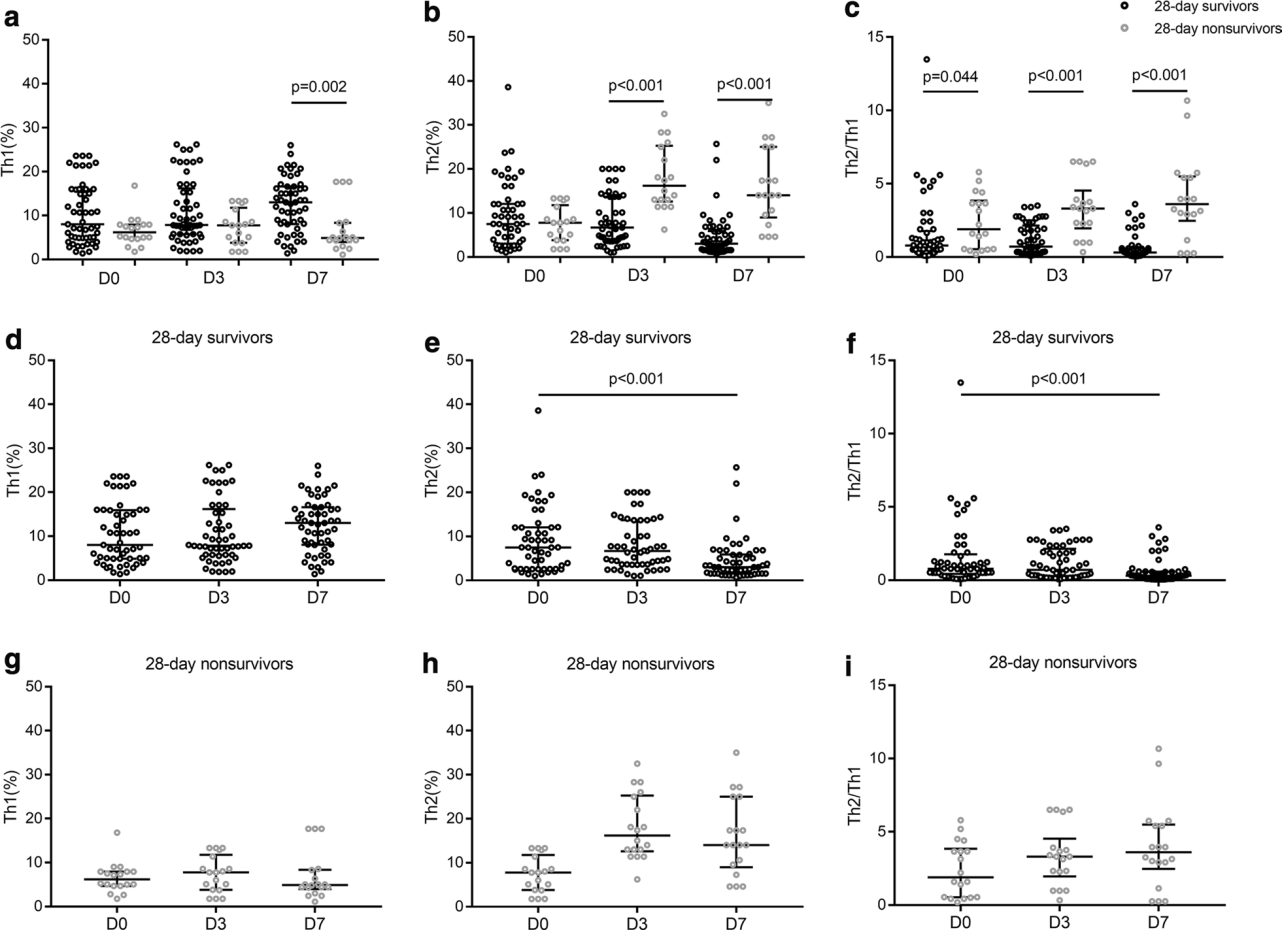
Values of Th1, Th2 and Th2/Th1 in community-acquired severe sepsis cohort stratified by 28-day survival status. Values of Th1 (**a**), Th2 (**b**) and Th2/Th1 (**c**) at different time points in community-acquired severe sepsis cohort stratified by 28-day survival status on D0, D3 and D7. The black scatter dot plots stand for 28-day survivors; the gray scatter dot plots stand for 28-day non-survivors; lines presented median value and quartiles. Values of Th1 (**d**), Th2 (**e**) and Th2/Th1 (**f**) on D0, D3 and D7 among 28-day survivors; Th1 (**g**), Th2 (**h**) and Th2/Th1 (**i**) on D0, D3 and D7 among 28-day non-survivors

Compared to survivors, non-survivors hold significantly higher Th2 populations and levels of serum IL-4 and IL-10 on D3 and D7 as well as higher Th2/Th1 than that of 28-day non-survivors on D0 (p = 0.044), D3 (p < 0.001) and D7 (p < 0.001), showing a sign of a more pronounced anti-inflammatory host response with Th2 dominance (Fig. 2 and Additional file 3: Table S1). However, no significant correlation was found between Th1 population and INF-γ levels. In terms of Th2 populations and plasma IL-4 levels, R square of correlation is less than 0.5 with a significant p value on D3 and D7 (Additional file 2: Figure S2).

By univariate analysis, populations of Th1 on D7, Th2 on D0, D3 and D7 and values of Th2/Th1 on D3 and D7 as well as alterations of Th2 within 7 days and Th2/Th1 within 3 days and 7 days were identified to be associated with 28-day prognosis (see Additional file 3: Table S2).

**Fig. 3:**
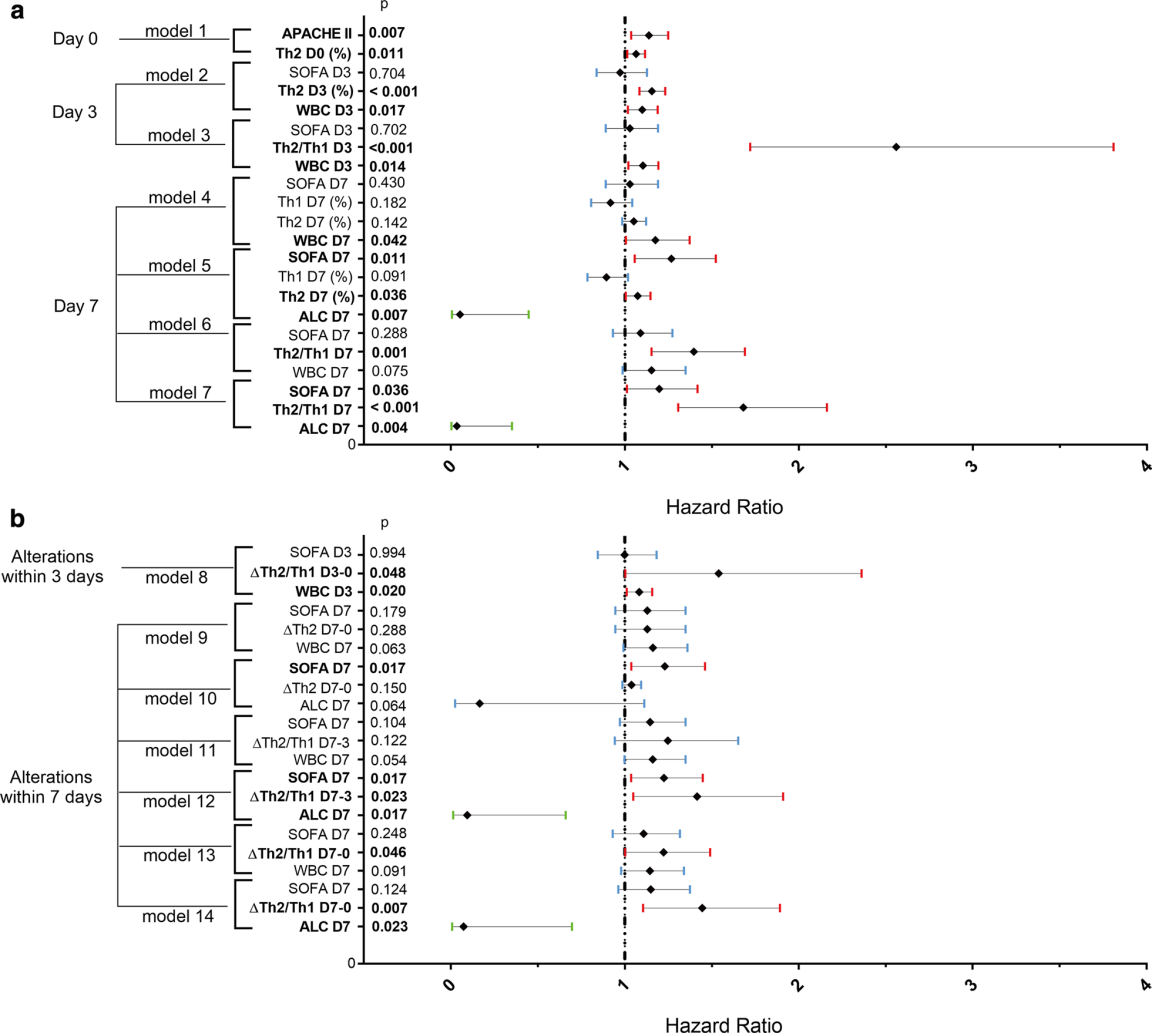
Death hazard ratios in cox-proportional hazards models. **a** Hazard ratios for severity scores, absolute values of peripheral blood cell count and T helper population. Significant risk factors are in red; Factors without significance are in blue; Significant protective factors are in green. **b** Hazard ratios for severity scores, alterations of peripheral blood cell count and T helper population within study period. Significant risk factors are in red; Factors without significance are in blue; Significant protective factors are in green. APACHE is for acute physiology and chronic health evaluation; SOFA is for sequential organ failure assessment; WBC is for white blood count; ALC is for absolute lymphocyte count; ΔTh2/Th1 D7-3 is change of Th2/Th1 from D7 to D3; ΔTh2/Th1 D7-3 is change of Th2/Th1 from D7 to D3. Significant associations with p < 0.05 are in bold

The variables of severity scores, inflammatory and immune indicators of peripheral blood cell count and T helper populations identified above associated with 28-day mortality entered different cox-proportional hazards models. Finally, fourteen specific cox models were conducted for including the variables that had no interaction with the others on D0, D3 and D7 respectively. The p value and HRs with 95% CI were illustrated in Fig. 3. It demonstrated that APACHE II score at admission, SOFA score on D7, Th2 populations on D0, D3 and D7, and Th2/Th1 on D3 and D7 as well as its increase to D3 and D7 from enrollment were independently associated with risk of 28-day mortality while ALC on D7 could be protective for 28-day prognosis.

**Fig. 4:**
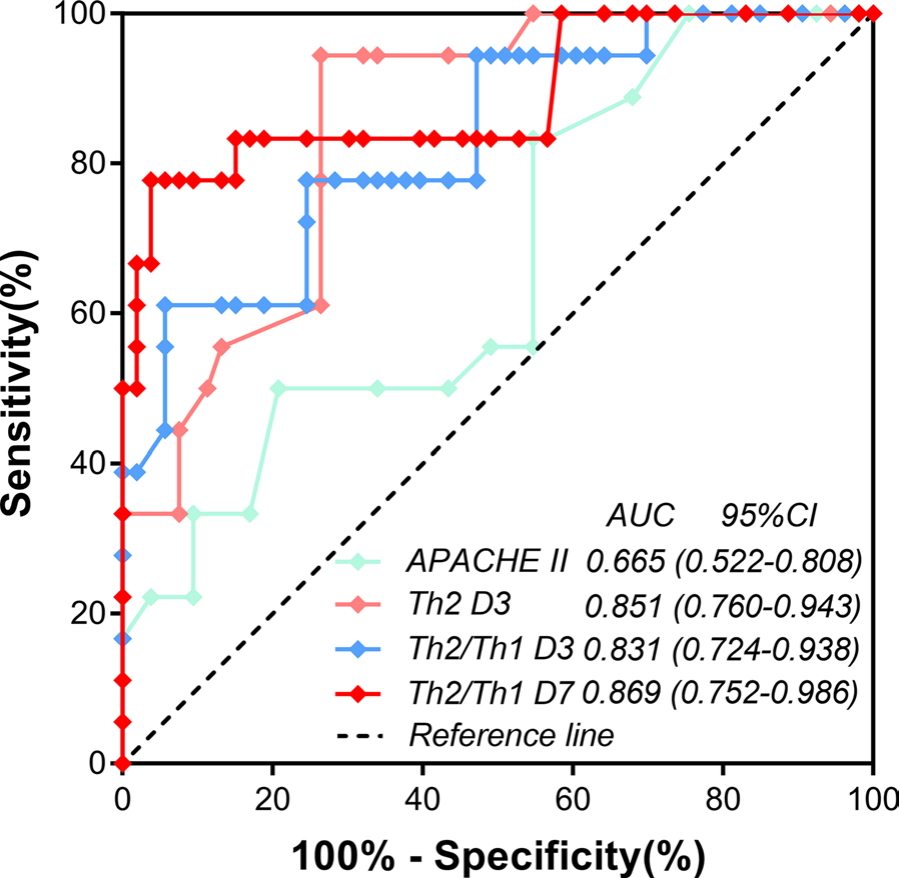
Receiver operating characteristic (ROC) curves. The area under curves (AUCs) with 95% confidence interval (CI) were presented with ROC curves. APACHE is for acute physiology and chronic health evaluation; AUC is for area under curve; CI is for confidence interval; Th is for T helper

More importantly, using the ROC curves to predict 28-day prognosis, we observed that Th2/Th1 on D3 and D7 demonstrated relatively high AUCs of 0.831 and 0.869 (Fig. 4). The best cut-off values of Th2/Th1 to predict 28-day mortality were 2.95 on D3 and 2.74 on D7, which hold a sensitivity of 66.1% and 75.0% as well as a specificity of 92.5% and 95.1%, respectively (Additional file 3: Table S3).

### Associations with inflammatory indicators, organ dysfunction, ICU-acquired infections and 28-day prognosis of Th2/Th1 alterations

**Fig. 5:**
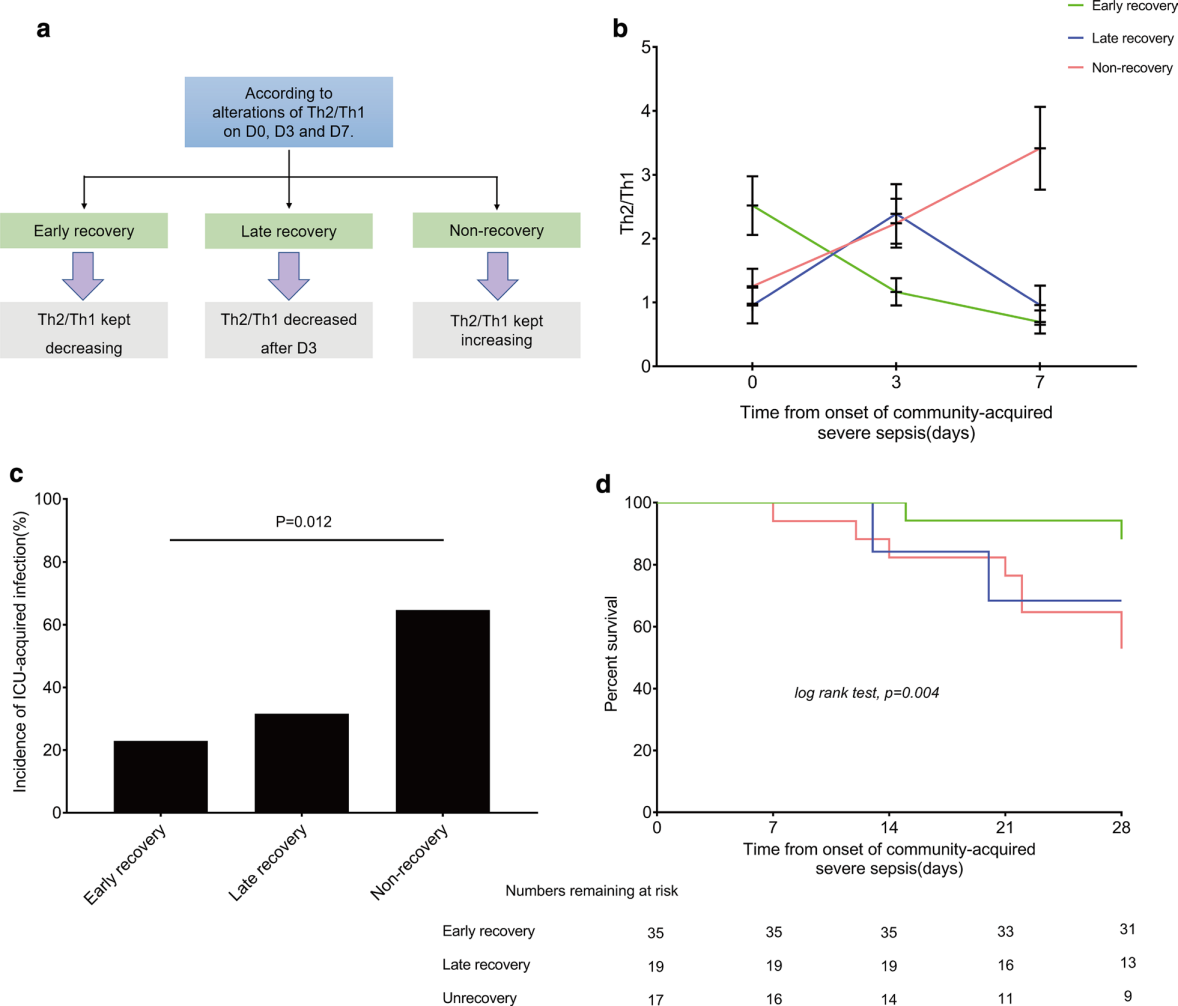
The persistent increasing of Th2/Th1 was associated with occurrence of ICU-acquired infections and 28-day death. **a** Depicts stratification by Th2/Th1 alterations. **b** Values of Th2/Th1 in subgroups. **c** Incidence of ICU-acquired infections of patients (n = 71) in subgroups. **d** Depicts survival curves of patients (n = 71) with community-acquired severe sepsis in subgroups

Patients were divided into subgroups based on alterations of Th2/Th1 (Fig. 5a). The early recovery group showed a decreasing trend of Th2/Th1 values with 2.51 ± 0.46, 1.11 ± 0.22 and 0.78 ± 0.19 while those in late recovery group were 0.95 ± 0.28, 2.39 ± 0.46 and 0.96 ± 0.31 and those in the non-recovery group were 1.26 ± 0.27, 2.33 ± 0.41 and 3.52 ± 0.76 on D0, D3 and D7, respectively (Fig. 5b). As the baseline characteristics of patients in subgroups showed (Table 2), patients in early recovery group had lowest SOFA scores on D3.

All patients have obvious systemic inflammatory responses on admission (Additional file 2: Figure S3). Patients in late recovery group had higher WBC than those in early recovery at the beginning. During the following week, lymphocyte counts recovered in early recovery group and were significantly higher than those of late recovery group on D3 (p = 0.009). Patients in late recovery and non-recovery groups were presented with higher HR on D7 than that in early recovery group. It was demonstrated a higher risk of AGI in late recovery and non-recovery groups compared to early recovery (OR 3.07, 95% CI 1.32–7.14, p = 0.006; OR 2.745, 95% CI 1.132–6.657, p = 0.023, respectively, see Additional file 3: Table S4).

Patients with non-recovery of Th2/Th1 had a higher incidence of ICU-acquired infections compared with those with early recovery (64.7% vs. 22.9%, p = 0.012, Fig. 5c). In addition, there was a significant difference of survival curves among subgroups (Fig. 5d). Patients with Th2/Th1 non-recovery had the highest mortality (47.1%), followed by 31.6% of late recovery of Th2/Th1, and the lowest mortality of 11.8% in early recovery group.

## Discussion

This study shows for the first time the dynamic and consecutive evaluation of peripheral T helper lymphocyte subpopulations in immunocompetent patients upon community-acquired severe sepsis onset. These data will support the concepts of early adaptive immune suppression with the imbalanced Th2/Th1 and its persistent existence was associated with poor outcome in severe sepsis.

Th2 dominance in T subpopulations in sepsis or severe sepsis was proved in previous studies among relatively heterogeneous populations, such as post traumatic patient [22] or mixed patients of hospital- and community-acquired sepsis [14]. For immunosuppression linking to hospitalization and trauma, change of T helper cell subset in context of severe sepsis needed further discussions. In our study, we focused on the relatively homogeneous population: previously immunocompetent patients upon onset of community-acquired severe sepsis within 24 h to clarify the causality relationship of alterations of T helper cell subset and severe sepsis.

In this study, we enrolled 71 patients with community-acquired severe sepsis with recordable sepsis-induced organ dysfunction within 24 h. Most enrolled septic patients were from the emergency department and had an older average age (71.2 years) than reported in a previous study [17]. The most frequent infectious focus was pulmonary (70.4%) which is the same as reported in most studies [23–28]. The mortality rates in our study appeared higher (25.3%) than what has been reported in other studies [24–27], which might be attributed to the potential role of immune-senescence following older average age of enrolled population in our study.

Previous studies have reported alterations of T helper lymphocyte in shifts towards Th2 in post trauma patients who had developed sepsis, which was associated with poor prognosis [22]. Our study demonstrated that Th2/Th1 and Th2 cell populations were up-regulated upon severe sepsis onset in immunocompetent patients. In addition, septic patients who died had an immune phenotype characterised by persistently higher Th2/Th1 with Th2 dominance. Along with the proinflammatory Th1 cells-driven response, almost all the septic patients had an obvious inflammatory response with elevated temperature, heart rates and white blood cell counts while only non-survivors held persistent Th2 dominance and non-recovery of Th2/Th1. Th2 population and Th2/Th1 ratios were independently associated with 28-day mortality, indicating a suppressed state of the adaptive immune system in patients who died, which was partly consistent with a previous study [29].

Subgroup analysis in septic cohort showed that patients without Th2/Th1 recovery were at risk of 28-day poor outcomes. There are several plausible explanations as following. Differentiation of surviving CD4 T cells from a pro-inflammatory Th1 cell phenotype to an anti-inflammatory Th2 cell phenotype, which is proposed to be a “protective” phenotype [30–32], turned out to be an impaired antimicrobial response to invading pathogens. There was also a strong trend towards increasing secondary infections in persistently rising Th2/Th1 subgroups. It is possible that increasing susceptibility to secondary infections contributes to the higher mortality in patients with the persistent increase of Th2/Th1. These findings are particularly striking because the optimal host response to sepsis should be to avoid the Th2 cell shift during CD4+ T lymphocyte differentiation and thereby augment Th1 cells.

The findings among subgroups stratified by dynamic Th2/Th1 alterations have important clinical implications, including high-risk patients’ identification and individually targeted therapy to improve sepsis outcomes. Previous studies on INF-γ and IL-4 have shown elevated serum and plasma levels to be associated with the increase of peripheral Th1 and Th2 populations [33]. However, correlation of plasma levels of INF-γ and IL-4 and T helper populations of Th1 and Th2 was not apparent in our study, which might be attributed to that plasma cytokines originate from various immune cells. Despite of taking at least 7 h for Th1 and Th2 measurements by flow cytometry, it is a feasible method for evaluating T cell status. Particularly for septic patients with rapid and severe onset, it is worth evaluating T cell subpopulations and identifying which part the uncontrolled and imbalanced host response originates from. More than a single value at a given time point, the dynamic alteration of Th2/Th1 would be strongly recommended to predict risk of ICU-acquired infection and death.

Our strengths lie in the study population selection and study period setting as well. By including only community-acquired severe sepsis patients without past histories affecting the immune system in septic cohort, we aimed to avoid bias by chronically hospitalization or other underlying impacts on immunological phenotypes. Previous studies have demonstrated independent associations between immunological indicators and poor outcome [34–39], but most have largely neglected the potential role of time since sepsis onset. The time of diagnosis or enrolment does not equal to the onset, which might cover the interaction between sepsis process and immune status. To explore the effect of severe sepsis on T helper subpopulations upon its onset, we presented data from previously immunocompetent patients with recordable manifestation or laboratory findings of sepsis-induced organ dysfunction within 24 h, which guaranteed inclusion in the relatively early stage as possible. Unlike the hypothesis of the inflammatory response coming first and immunosuppression following [2, 32], this study with an imbalanced Th2/Th1 as an immunosuppression indicator in severe sepsis supports the concepts that immunosuppression could occur in early sepsis.

There are several potential limitations. This was a single-centre study. Data collection was limited to variables available during usual care and resulted in an imbalance between groups. Besides, among 338 severe sepsis patients admitted to our centre during 2-year study period, most were excluded for hospital-acquired sepsis, diagnosis of tumour and receipt of chemotherapy or corticosteroid, resulting in enrolling 71 patients, which might bring enrolment bias. Another limitation of this study is that the exact time of severe sepsis onset could not be precisely determined, though we have defined the onset to be the time recordable manifestation or laboratory findings of sepsis-induced organ dysfunction, which was determined by the treating physician and may mask interpretation of timing in this study. Thirdly, T helper subpopulation at the cellular level could only reflect one aspect of immune system, which was focused on in our study and other immune cells like Th17 cells and indicators like related special transcription factors and cytokines might be included in the comprehensive strategy to evaluate the immune status regarding to sepsis.

## Conclusions

Th2/Th1 value is markedly up-regulated upon onset of community-acquired severe sepsis among previously immunocompetent patients and its persistently dynamic increase is associated with ICU-acquired infection and death within 28 days. In the clinical setting, a persistently increasing Th2/Th1 in septic patients should prompt clinicians to re-evaluate patients’ response to therapy and assess for the presence of new or untreated infections. In the future, dynamic measurements of Th2/Th1 will help stratify patients when setting clinical criteria for an adaptive immune-targeted therapy in a time-dependent manner.

## Additional files

**Additional file 1.** Procedure of T subpopulations measurements.

**Additional file 2: Figure S1.** Flowchart of included and excluded severe sepsis patients. **Figure S2.** Correlation analysis of T helper populations and plasma cytokines. **A, B** and **C** shows the correlation analysis of Th1 population and plasma INF-γ levels on Day 0, Day 3 and Day 7, respectively. **D, E** and **F** depicts correlation analysis of Th2 population and plasma IL-4 on Day 0, Day 3 and Day 7, respectively. Th is for T helper; INF is for interferon; IL is for interleukin. **Figure S3.** Inflammatory and immune indicators in subgroups stratified by dynamic alterations of Th2/Th1. **A** depicts mean values of HR and T with standard deviation on D0, D3 and D7. **B** and **C** show mean values with mean squared error of PCT and hs-CRP on D0, D3 and D7. **D** and **E** present median WBC and ALC values with interquartile range on D0, D3 and D7.

**Additional file 3: Table S1.** Concentrations of plasma cytokines in peripheral blood. **Table S2.** Univariate cox regression analysis of variables associated with the 28-day mortality. **Table S3.** Diagnostic ability of various variables to predict 28-day prognosis, presenting with AUC and best cut-off value with its sensitivity and specificity. **Table S4.** Complicated organ dysfunctions within 28 days in subgroups stratified by dynamic alterations of Th2/Th1.

## Abbreviations

AGI: acute gastrointestinal injury
AKI: acute kidney injury
ALC: absolute lymphocyte cell
APACHE: acute physiology and chronic health evaluation
ARDS: acute respiratory distress syndrome
AUC: areas under the curves
CI: confidence interval
ESICM: European Society of Intensive Care Medicine
Hs-CRP: hypersensitive C-reactive protein
ICU: intensive care unit
IFN: interferon
IL: interleukin
IQR: interquartile range
KDIGO: kidney disease improving global outcomes
PASS: Power Analysis and Sample Size
PCT: procalcitonin
ROC: receiver-operating characteristic
SD: standard deviation
SEM: mean squared error
SOFA: sequential organ failure assessment
Th: T helper
WBC: white blood cell
