# Supplementary material for: Early and dynamic alterations of Th2/Th1 in previously immunocompetent patients with community-acquired severe sepsis: a prospective observational study
Source: J Transl Med. 2019 Feb 27;17:57. doi: 10.1186/s12967-019-1811-9 (PMC6391803; doi:10.1186/s12967-019-1811-9)
Supplement: Supplementary file 1 — Additional file 1. Procedure of T subpopulations measurements. [file 12967_2019_1811_MOESM1_ESM.docx]

**Procedure of T subpopulations measurements.**

**A. Procedure of T helper 1 and T helper 2 cells measurements**

Peripheral whole blood of 100μl was stimulated for 4 hours with 2 u/mL leukocyte activation cocktail (BD Pharmingen™, USA) at 37°C and 5% CO2. Upon harvest, erythrocytes were lysed by red blood cell lysis buffer, and cells were then washed twice using PBS and resuspended to 1 × 10^6^ cells in a 100μl BD FACS™ Lysing Solution. Cells were surface stained with mouse anti-Human CD8a-APC (BD Pharmingen™, USA), mouse anti-Human CD3-FITC (BD Pharmingen™, USA) at room temperature in the dark and then fixed and permeabilized using intracellular Fixation & Permeabilization (BD Pharmingen™, USA). Following that, cells were incubated with PE-conjugated mouse anti-Human INF γ (BD Pharmingen™, USA) and PE-conjugated mouse anti-Human IL-4(BD Pharmingen™, USA). Samples were run on a flow cytometer (MACSQuantTM, Germany). Data were analysed using Flowjo software (FLOWJO, LLC, USA). The frequencies of Th1 (CD3^+^CD8^-^IFN-γ^+^), Th2 (CD3^+^CD8^-^IL-4^+^) cells were expressed as a percentage of CD3^+^ CD8^－^T cells by sequential gating for lymphocytes. CD4 positive T cells equal lymphocytes with CD3 positive and CD8 negative cells.

**B. Procedure of regulatory T cells measurements**

Peripheral whole blood of 100μl was surface stained with mouse anti-Human CD25-APC (BD Pharmingen™, USA), mouse anti-Human CD4-FITC (BD Pharmingen™, USA) at room temperature in the dark and then erythrocytes were lysed by red blood cell lysis buffer. Cells were then washed twice using PBS and resuspended to 1 × 10^6^ cells in a 100μl BD FACS™ Lysing Solution. Following that, the samples were fixed and permeabilized using intracellular Fixation & Permeabilization (BD Pharmingen™, USA) and then were incubated with PE-conjugated mouse anti-Foxp3 (BD Pharmingen™, USA) added as recommended. Samples were run on a flow cytometer (MACSQuantTM, Germany). Data were analysed using Flowjo software (FLOWJO, LLC, USA). The frequency of regulatory T cells was identified as a percentage of positive expression of CD4, CD25 and Foxp3 in positive expression of CD4 cells.
