# Supplementary material for: Early and dynamic alterations of Th2/Th1 in previously immunocompetent patients with community-acquired severe sepsis: a prospective observational study
Source: J Transl Med. 2019 Feb 27;17:57. doi: 10.1186/s12967-019-1811-9 (PMC6391803; doi:10.1186/s12967-019-1811-9)
Supplement: Supplementary file 2 — Additional file 2: Figure S1. Flowchart of included and excluded severe sepsis patients. Figure S2. Correlation analysis of T helper populations and plasma cytokines. A, B and C shows the correlation analysis of Th1 population and plasma INF-γ levels on Day 0, Day 3 and Day 7, respectively. D, E and F depicts correlation analysis of Th2 population and plasma IL-4 on Day 0, Day 3 and Day 7, respectively. Th is for T helper; INF is for interferon; IL is for interleukin. Figure S3. Inflammatory and immune indicators in subgroups stratified by dynamic alterations of Th2/Th1. A depicts mean values of HR and T with standard deviation on D0, D3 and D7. B and C show mean values with mean squared error of PCT and hs-CRP on D0, D3 and D7. D and E present median WBC and ALC values with interquartile range on D0, D3 and D7. [file 12967_2019_1811_MOESM2_ESM.docx]

**
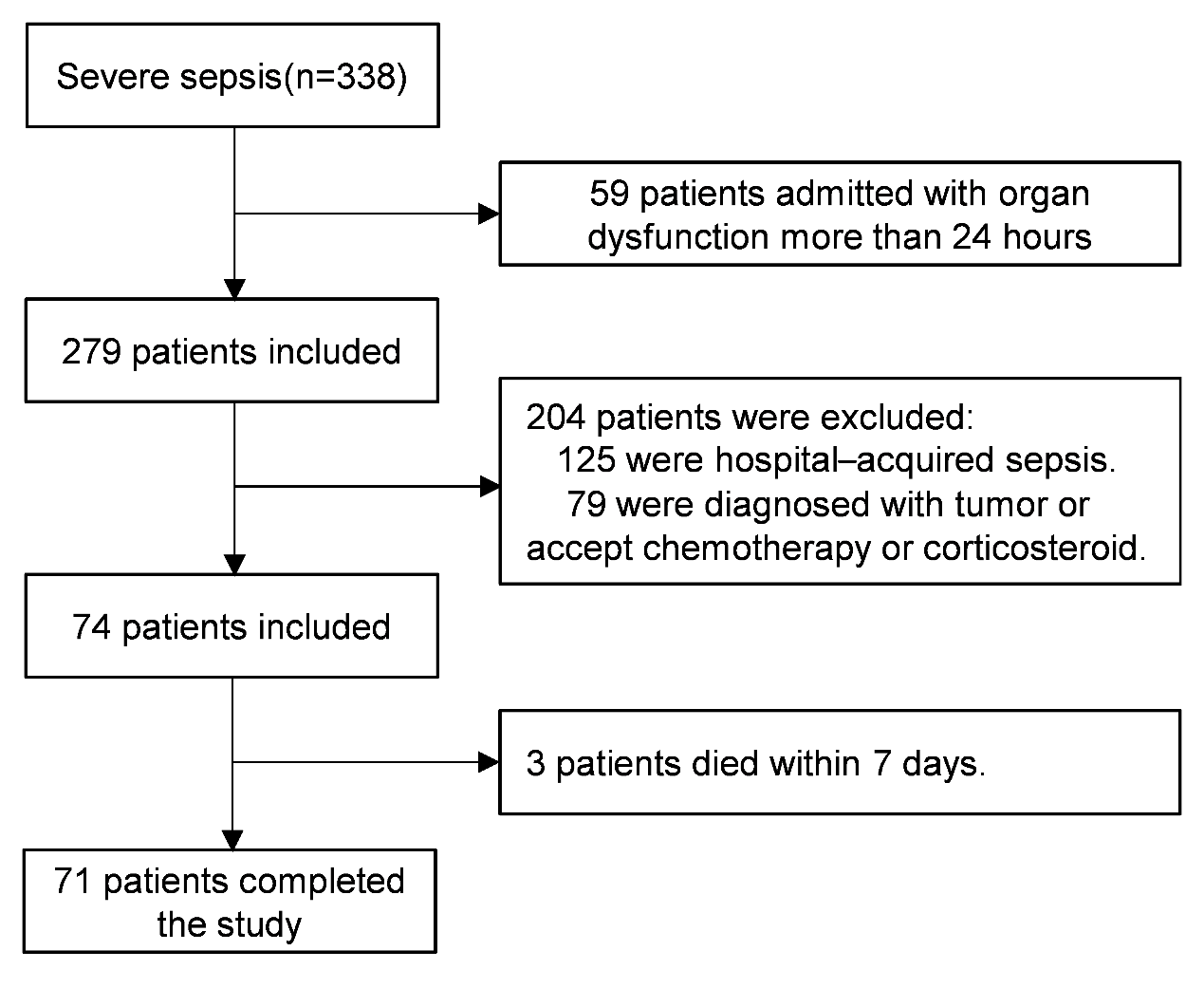
**

**Figure S1** Flowchart of included and excluded severe sepsis patients.


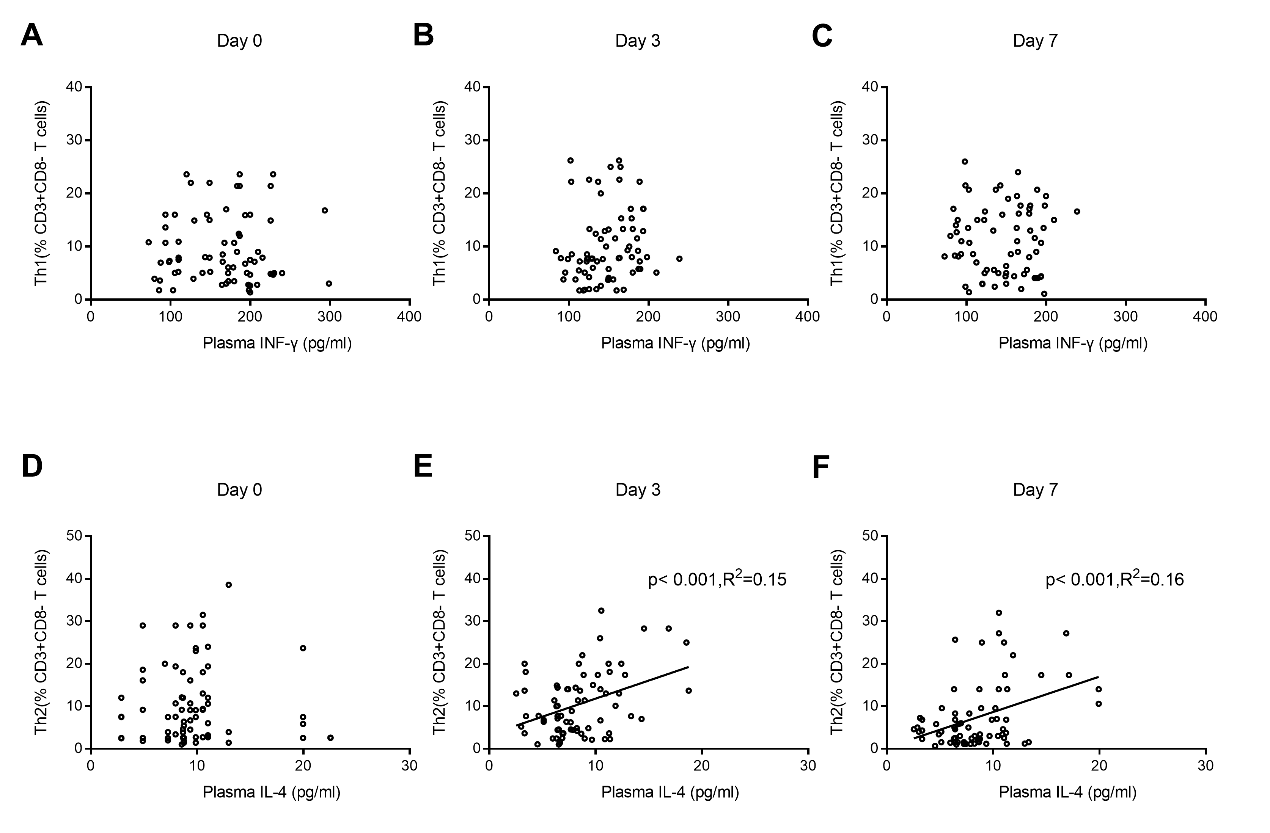


**Figure S2** Correlation analysis of T helper populations and plasma cytokines. **A, B** and **C** shows the correlation analysis of Th1 population and plasma INF-γlevels on Day 0, Day 3 and Day 7,respectively. **D, E** and **F** depicts correlation analysis of Th2 population and plasma IL-4 on Day 0, Day 3 and Day 7,respectively. Th is for T helper; INF is for interferon; IL is for interleukin.


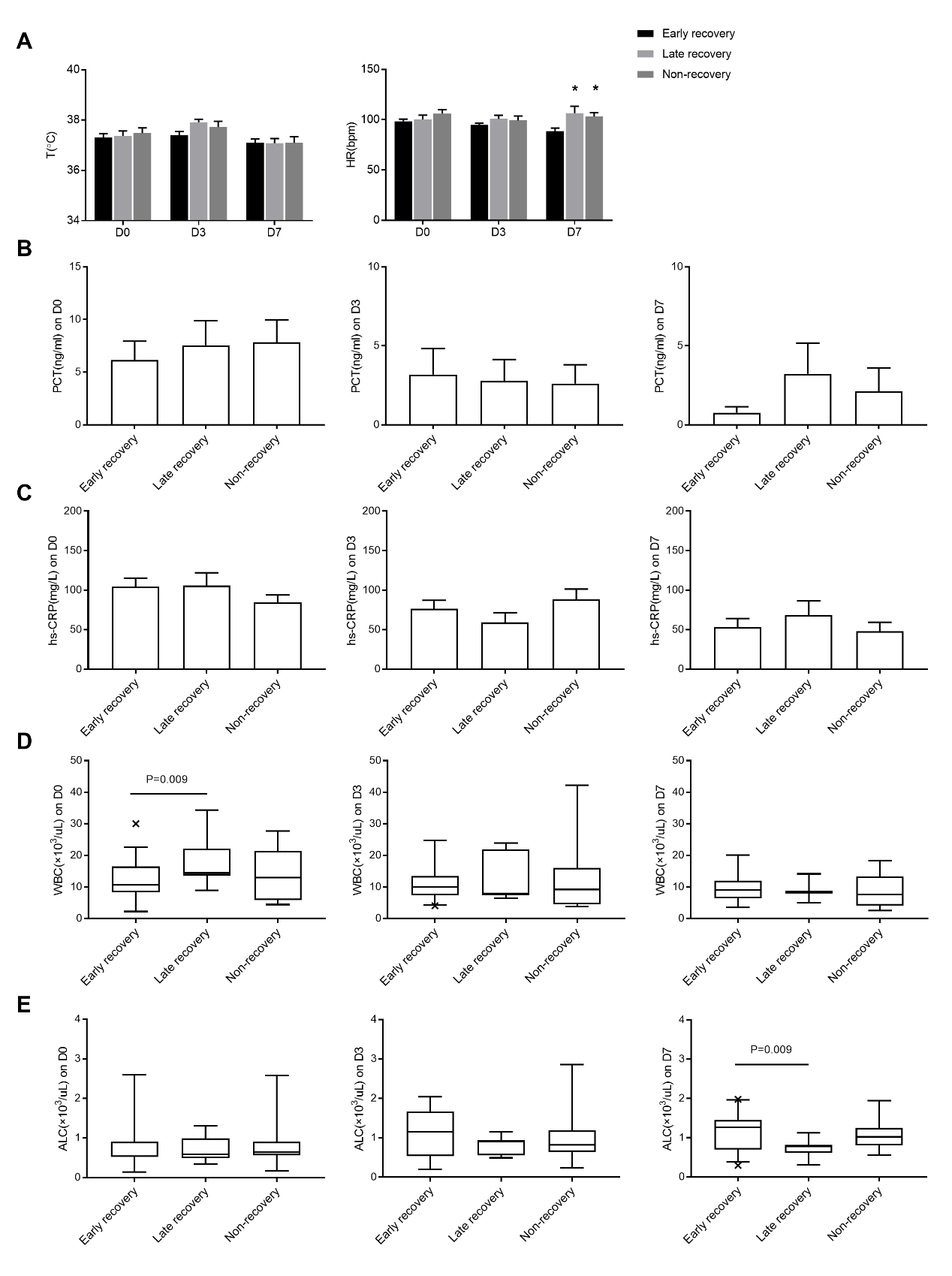


**Figure S3** Inﬂammatory and immune indicators in subgroups stratified by dynamic alterations of Th2/Th1. **A** depicts mean values of HR and T with standard deviation on D0, D3 and D7. **B** and **C** show mean values with mean squared error of PCT and hs-CRP on D0, D3 and D7. **D** and **E** present median WBC and ALC values with interquartile range on D0, D3 and D7.
