# Supplementary material for: Early and dynamic alterations of Th2/Th1 in previously immunocompetent patients with community-acquired severe sepsis: a prospective observational study
Source: J Transl Med. 2019 Feb 27;17:57. doi: 10.1186/s12967-019-1811-9 (PMC6391803; doi:10.1186/s12967-019-1811-9)
Supplement: Supplementary file 3 — Additional file 3: Table S1. Concentrations of plasma cytokines in peripheral blood. Table S2. Univariate cox regression analysis of variables associated with the 28-day mortality. Table S3. Diagnostic ability of various variables to predict 28-day prognosis, presenting with AUC and best cut-off value with its sensitivity and specificity. Table S4. Complicated organ dysfunctions within 28 days in subgroups stratified by dynamic alterations of Th2/Th1. [file 12967_2019_1811_MOESM3_ESM.docx]

**Table S1. Concentrations of plasma cytokines in peripheral blood.**

| **Concentration. pg/ml**  (mean±SD) | **Healthy control**  **n=7** | **ICU control**  **n=7** | **Community-acquired severe sepsis**  **n=71** | | | |
| --- | --- | --- | --- | --- | --- | --- |
|  |  |  | **overall**  **n=71** | **28-day survivors**  **n= 53(74.6%)** | **28-day non-survivors**  **n=18(25.4%)** | **P** |
| Day 0 |  |  |  |  |  |  |
| INF-γ | 75.6 ± 8.08^*^ | 193.0 ± 42.6 | 166.4 ± 51.2 | 162.2 ± 52.0 | 178.8 ± 48.0 | NS |
| IL-4 | 1.9 ± 0.5^*^ | 3.6 ± 2.0^*^ | 9.6 ± 4.2 | 9.2 ± 3.9 | 10.5 ± 5.0 | NS |
| IL-10 | 2.4 ± 0.8^*^ | 5.9 ± 2.2^*^ | 8.0 ± 2.7 | 7.9 ± 2.7 | 8.5 ± 2.7 | NS |
| Day 3 |  |  |  |  |  |  |
| INF-γ |  |  | 148.7 ± 33.0 | 150.7 ± 33.4 | 143.0 ± 31.8 | NS |
| IL-4 |  |  | 8.5 ± 3.4 | 7.9 ± 3.0 | 10.2 ± 4.1 | 0.012 |
| IL-10 |  |  | 7.2 ± 2.3 | 6.7 ± 2.1 | 8.6 ± 2.3 | 0.002 |
| Day 7 |  |  |  |  |  |  |
| INF-γ |  |  | 145.1 ± 39.6 | 141.6 ± 40.1 | 155.4 ± 37.2 | NS |
| IL-4 |  |  | 8.5 ± 3.7 | 7.7 ± 2.6 | 11.1 ± 5.1 | 0.004 |
| IL-10 |  |  | 6.8 ± 2.1 | 6.4 ± 2.0 | 7.9 ± 2.2 | 0.023 |

SD is for standard deviation; NS is for not significant; INF is for Interferon; IL is for interleukin; * is for significant difference with p < 0.05 compared to community-acquired severe sepsis.

**Table S2. Univariate cox regression analysis of variables associated with the 28-day mortality.**

| **Variables** | **P** | **Exp(B)** |
| --- | --- | --- |
| Age | 0.950 | 0.999 |
| gender | 0.138 | 2.014 |
| Comorbidities |  |  |
| Hypertension | 0.952 | 1.029 |
| Cardiovascular dysfunction | 0.931 | 1.043 |
| Diabetes | 0.264 | 1.716 |
| Cerebrovascular disease | 0.628 | 0.785 |
| Chronic renal dysfunction | 0.867 | 0.842 |
| Severity score |  |  |
| **APACHE II** | **0.008** | **1.128** |
| SOFA D0 | 0.387 | 1.069 |
| **SOFA D3** | **0.025** | **1.168** |
| **SOFA D7** | **0.005** | **1.254** |
| T cell populations |  |  |
| Th1 D0 | 0.053 | 0.908 |
| **Th2 D0** | **0.015** | **1.057** |
| Th2/Th1 D0 | 0.176 | 1.107 |
| Treg D0 | 0.863 | 1.020 |
| Th1 D3 | 0.070 | 0.917 |
| **Th2 D3** | **<0.001** | **1.148** |
| **Th2/Th1 D3** | **<0.001** | **2.396** |
| Treg D3 | 0.082 | 0.632 |
| **Th1 D7** | **0.003** | **0.863** |
| **Th2 D7** | **<0.001** | **1.123** |
| **Th2/Th1 D7** | **<0.001** | **1.641** |
| Treg D7 | 0.595 | 1.031 |
| Peripheral blood cell counts |  |  |
| WBC D0 | 0.405 | 1.024 |
| ALC D0 | 0.128 | 0.329 |
| **WBC D3** | **0.005** | **1.102** |
| ALC D3 | 0.117 | 0.471 |
| **WBC D7** | **0.001** | **1.179** |
| **ALC D7** | **0.013** | **0.150** |
| Inflammatory indicators |  |  |
| PCT D0 | 0.124 | 0.948 |
| hs-CRP D0 | 0.122 | 0.993 |
| PCT D3 | 0.549 | 0.966 |
| hs-CRP D3 | 0.668 | 0.998 |
| PCT D7 | 0.965 | 1.002 |
| hs-CRP D7 | 0.472 | 0.995 |
| Plasma cytokines |  |  |
| INF-γ D0 | 0.237 | 1.007 |
| INF-γ D3 | 0.391 | 0.993 |
| INF-γ D7 | 0.206 | 1.009 |
| IL-4 D0 | 0.286 | 1.069 |
| **IL-4 D3** | **0.020** | **1.218** |
| **IL-4 D7** | **0.003** | **1.316** |
| IL-10 D0 | 0.361 | 1.098 |
| **IL-10 D3** | **0.003** | **1.518** |
| **IL-10 D7** | **0.006** | **1.495** |
| Alterations of T cell populations within study period | | |
| ΔTh1 D3-0 | 0.937 | 1.004 |
| ΔTh2 D3-0 | 0.092 | 1.041 |
| **ΔTh2/Th1 D3-0** | **0.006** | **1.562** |
| ΔTreg D3-0 | 0.186 | 0.875 |
| ΔTh1 D7-3 | 0.196 | 0.947 |
| ΔTh2 D7-3 | 0.336 | 1.037 |
| **ΔTh2/Th1 D7-3** | **0.006** | **1.461** |
| ΔTreg D7-3 | 0.181 | 1.072 |
| ΔTh1 D7-0 | 0.290 | 0.959 |
| **ΔTh2 D7-0** | **0.031** | **1.048** |
| **ΔTh2/Th1 D7-0** | **<0.001** | **1.403** |
| ΔTreg D7-0 | 0.649 | 1.027 |
| Alterations of Peripheral blood cell counts within study period | | |
| ΔWBC D3-0 | 0.158 | 0.952 |
| ΔALC D3-0 | 0.887 | 1.055 |
| ΔWBC D7-3 | 0.983 | 0.999 |
| ΔALC D7-3 | 0.647 | 0.834 |
| ΔWBC D7-0 | 0.188 | 0.954 |
| ΔALC D7-0 | 0.745 | 0.884 |

APACHE is for acute physiology and chronic health evaluation; SOFA is for sequential organ failure assessment; Th is for T helper; Treg is for regulatory T cells; WBC is for white blood cell; ALC is for absolute lymphocyte count; PCT is for procalcitonin; hs-CRP is for hyper-sensitive C-reactive protein; INF is for interferon; IL is for interleukin; ΔD3-0 is change from D3 to D0; ΔD7-0 is change from D7 to D0; ΔD7-3 is change from D7 to D3. Significant associations with p<0.05 are in bold.

**Table S3.** **Diagnostic ability of various variables to predict 28-day prognosis, presenting with AUC and best cut-off value with its sensitivity and specificity^*^**

| **Variables** | **AUC** | **P** | **95%**  **confidence interval** | **Best cut-off value** | **Sensitivity** | **Specificity** |
| --- | --- | --- | --- | --- | --- | --- |
| D0 |  |  |  |  |  |  |
| **APACHE II** | **0.665** | **0.037** | **0.522-0.808** | **23.5** | **0.500** | **0.792** |
| SOFA | 0.599 | 0.212 | 0.453-0.745 | - | - | - |
| Th1 | 0.373 | 0.108 | 0.240-0.505 | - | - | - |
| Th2 | 0.610 | 0.167 | 0.448-0.771 | - | - | - |
| Th2/Th1 | 0.666 | 0.036 | 0.521-0.811 | 1.35 | 0.667 | 0.736 |
| WBC | **0.575** | 0.341 | 0.412-0.739 | 21.45 | 0.389 | 0.925 |
| ALC | 0.385 | 0.148 | 0.256-0.515 | - | - | - |
| D3 |  |  |  |  |  |  |
| SOFA | 0.656 | 0.086 | 0.487-0.825 | - | - | - |
| Th1 | 0.379 | 0.129 | 0.234-0.525 | - | - | - |
| **Th2** | **0.851** | **<0.001** | **0.760-0.943** | **10.75** | **0.944** | **0.736** |
| **Th2/Th1** | **0.831** | **<0.001** | **0.724-0.938** | **2.95** | **0.661** | **0.925** |
| **WBC** | **0.668** | **0.034** | **0.521-0.814** | 10.63 | 0.722 | 0.680 |
| ALC | 0.320 | 0.023 | 0.177-0.463 | - | - | - |
| D7 |  |  |  |  |  |  |
| SOFA | 0.719 | 0.011 | 0.564-0.874 | 7.5 | 0.750 | 0.780 |
| Th1 | 0.290 | 0.019 | 0.109-0.470 | - | - | - |
| **Th2** | **0.863** | **<0.001** | **0.758-0.968** | **7.07** | **0.800** | **0.800** |
| **Th2/Th1** | **0.869** | **0.000** | **0.752-0.986** | **2.74** | **0.750** | **0.951** |
| WBC | 0.668 | 0.051 | 0.491-0.844 | 13.35 | 0.563 | 0.902 |
| ALC | 0.305 | 0.023 | 0.146-0.463 | - | - | - |
| Alterations of T helper populations and peripheral blood cell counts | | | | | | |
| Th2 D3-0 | 0.656 | 0.050 | 0.479-0.833 | - | - | - |
| Th2 D7-0 | **0.681** | 0.023 | 0.508-0.854 | 1.70 | 0.667 | 0.830 |
| Th2 D7-3 | 0.552 | 0.513 | 0.389-0.714 | - | - | - |
| Th1 D3-0 | 0.506 | 0.942 | 0.354-0.657 | - | - | - |
| Th1 D7-0 | 0.350 | 0.059 | 0.191-0.509 | - | - | - |
| Th1 D7-3 | 0.369 | 0.098 | 0.229-0.509 | - | - | - |
| Th2/Th1 D3-0 | **0.742** | 0.002 | 0.597-0.887 | 0.23 | 0.778 | 0.755 |
| Th2/Th1 D7-3 | 0.581 | 0.309 | 0.392-0.769 | - | - | - |
| Th2/Th1 D7-0 | **0.735** | 0.003 | 0.582-0.888 | 0.27 | 0.667 | 0.906 |
| ALC D3-0 | 0.588 | 0.267 | 0.443-0.733 | - | - | - |
| ALC D7-0 | 0.544 | 0.579 | 0.398-0.690 | - | - | - |
| ALC D7-3 | 0.483 | 0.833 | 0.325-0.641 | - | - | - |
| WBC D3-0 | 0.377 | 0.122 | 0.231-0.523 | - | - | - |
| WBC D7-0 | 0.477 | 0.771 | 0.313-0.641 | - | - | - |
| WBC D7-3 | 0.496 | 0.958 | 0.327-0.665 | - | - | - |

^*^Only variables with p < 0.05 and AUC above 0.5 presented best cut-off value with its sensitivity and specificity. Variables with p < 0.05 and AUC above 0.75 were presented in bold; ROC receiver operating characteristic; AUC area under curve; APACHE is for Acute Physiology and Chronic Health Evaluation; SOFA is for sequential organ failure assessment; WBC is for white blood cell; ALC is for absolute lymphocyte count. D3-0 is change from D3 to D0; D7-0 is change from D7 to D0; D7-3 is change from D7 to D3.

**Table S4.** **Complicated organ dysfunctions within 28 days in subgroups stratified by dynamic alterations of Th2/Th1.**

|  | **Early recovery** | **Early recovery** | **Non-recovery** |
| --- | --- | --- | --- |
| **Complicated with** | **(n=35)** | **(n=19)** | **(n=17)** |
| ARDS, n (%) | 31(88.6) | 19(100) | 17(100) |
| OR (95%CI)  P | -  - | 1.129(1.002-1.272)  0.126 | 1.542(1.256-1.909)  0.147 |
| Circulatory shock, n (%) | 31(88.6) | 19(100) | 17(100) |
| OR (95%CI)  P | -  - | 1.129(1.002-1.272)  0.126 | 1.542(1.256-1.909)  0.147 |
| AKI, n (%) | 14(40) | 7(36.8) | 9(52.4) |
| OR (95%CI)  P | -  - | 0.921(0.451-1.883)  0.820 | 1.324(0.723-2.423)  0.378 |
| AGI, n (%) | 6(17.1) | 10(52·6) | 8(47.1) |
| OR (95%CI)  P | -  - | 3.070(1.32-7.14)  0.006 | 2.745(1.132-6.657)  0.023 |
| CNS dysfunction, n (%) | 4(11.4) | 6(31.6) | 5(29.4) |
| OR (95%CI)  P | -  - | 2.763(0.888-8.598)  0.069 | 2.574(0.791-8.377)  0.108 |

ARDS is for acute respiratory distress syndrome; OR is for odds ratio; AKI is for acute kidney injury; AGI is for acute gastrointestinal injury; CNS is for central nervous system.
